# Dimethyl Fumarate Protects Pancreatic Islet Cells and Non-Endocrine Tissue in L-Arginine-Induced Chronic Pancreatitis

**DOI:** 10.1371/journal.pone.0107111

**Published:** 2014-09-08

**Authors:** Lourdes Robles, Nosratola D. Vaziri, Shiri Li, Yuichi Masuda, Chie Takasu, Mizuki Takasu, Kelly Vo, Seyed H. Farzaneh, Michael J. Stamos, Hirohito Ichii

**Affiliations:** Departments of Surgery and Medicine, University of California Irvine, Irvine, CA, United States of America; University of Szeged, Hungary

## Abstract

**Background:**

Chronic pancreatitis (CP) is a progressive disorder resulting in the destruction and fibrosis of the pancreatic parenchyma which ultimately leads to impairment of the endocrine and exocrine functions. Dimethyl Fumarate (DMF) was recently approved by FDA for treatment of patients with multiple sclerosis. DMF's unique anti-oxidant and anti-inflammatory properties make it an interesting drug to test on other inflammatory conditions. This study was undertaken to determine the effects of DMF on islet cells and non-endocrine tissue in a rodent model of L-Arginine-induced CP.

**Methods:**

Male Wistar rats fed daily DMF (25 mg/kg) or vehicle by oral gavage were given 5 IP injections of L-Arginine (250 mg/100 g×2, 1 hr apart). Rats were assessed with weights and intra-peritoneal glucose tolerance tests (IPGTT, 2 g/kg). Islets were isolated and assessed for islet mass and viability with flow cytometry. Non-endocrine tissue was assessed for histology, myeloperoxidase (MPO), and lipid peroxidation level (MDA). *In vitro* assessments included determination of heme oxygenase (HO-1) protein expression by Western blot.

**Results:**

Weight gain was significantly reduced in untreated CP group at 6 weeks. IPGTT revealed significant impairment in untreated CP group and its restoration with DMF therapy (P <0.05). Untreated CP rats had pancreatic atrophy, severe acinar architectural damage, edema, and fatty infiltration as well as elevated MDA and MPO levels, which were significantly improved by DMF treatment. After islet isolation, the volume of non-endocrine tissue was significantly smaller in untreated CP group. Although islet counts were similar in the two groups, islet viability was significantly reduced in untreated CP group and improved with DMF treatment. *In vitro* incubation of human pancreatic tissue with DMF significantly increased HO-1 expression.

**Conclusion:**

Administration of DMF attenuated L-Arginine-induced CP and islet function in rats. DMF treatment could be a possible strategy to improve clinical outcome in patients with CP.

## Introduction

Chronic pancreatitis (CP) is a progressive inflammatory disorder that results in the destruction and fibrosis of the pancreatic parenchyma and its endocrine and exocrine dysfunctions. Although CP can develop from repeated attacks of AP, this is not the only mechanism implicated. The specific pathogenesis is uncertain and has thus resulted in a lack of progress in developing specific therapies [1], [2]. Data suggest that the incidence of CP continues to rise [3]. In structured questionnaires, patients with CP described themselves as living with a complex illness with a significant impact on their physical, social, and psychological wellbeing [4]. Likewise, financial problems are more frequent amongst patients with CP. In a United Kingdom series, 37% of CP patients were unemployed [5]. Moreover, the mortality rate appears to be higher in patient with long standing CP. At 5 years the survival rate is initially high at 97% however, this drops to between 45% and 63% at 20 years.

Currently, the main treatment for this debilitating disease is supportive care through nutrition and pain control. Dimethyl Fumarate (DMF) was recently approved by the Food and Drug Administration (FDA) for use in the treatment of patients with multiple sclerosis. The exact mechanism of action of DMF has yet to be clearly determined, however its unique antioxidant and inflammatory properties have been shown in experimental inflammatory conditions [6], [7], [8], [9], [10], [11].

The aim of the present study was to test the hypothesis that DMF treatment would protect islets and non-endocrine cells from oxidative stress and inflammation caused by chronic pancreatitis.

## Methods

### Chronic pancreatitis

This study was carried out in strict accordance with the Guidelines for the Care and Use of Laboratory Animals of the National Institutes of Health. The protocol was approved by Institutional Animal Care and Use Committee of University of California, Irvine (Permit Number: 2012–3069). Animals were purchased from Charles River (Wilmington, MA). L-arginine and DMF were purchased from Sigma (St. Louis, MO). Male Wistar rats (250–300 g) were fed ad libitum on a standard diet with free access to water and maintained on a 12 h light/dark cycle. The rats were divided into four groups: Control (n = 3), DMF alone treated (n = 3), L-arginine + DMF-treated (n = 6), and L-arginine + vehicle- treated rats (n = 6).

All surgical procedures were performed under isoflurane anesthesia (1–5%) (Phoenix, St. Joseph, MO) adjusted to achieve no movement with paw prick testing. All efforts were made to minimize suffering. Experimental animals were given oral DMF (25 mg/kg) dissolved in methyl cellulose and fed via oral gavage 24 hours prior to initiating pancreatitis and daily thereafter until the animals were sacrificed. The dose of DMF was based on previously published studies [12] and preliminary studies done in our laboratory. The healthy control rats as well as the subgroup of rats with L-Arginine induced CP were given methyl cellulose vehicle (0.08%, 2.5–3 ml/rat). Weekly body weight assessments (Denver Instrument, Bohemia, NY) and change in body weight from baseline were calculated.

### L-arginine

20% L-arginine was dissolved in normal saline and filtered through a syringe filter with pH adjusted to 7.0. The solution was administered to non-fasted rats in two intraperitoneal injections at a dose of 250 mg/100 g body weight, each injection separated by 1 hour. L-Arginine injections were given a total of 5 times three days apart (Days 1, 5, 9, 13, 17) and animals were sacrificed after 7 weeks (on day 54, 37 days after the last L-arginine injection) [13]. Animals, control and experimental, received Buprenorphine (Reckitt Benkiser, Richmond, VA) pain medication (0.01 mg/kg) IM twice a day for two days after each L-arginine injection. Animals had accesses to regular food and water and were not fasted prior to L-arginine injections. All efforts were made to minimize pain and suffering. Experimental animals continued to receive daily DMF via gavage along with a standard diet.

### Intraperitoneal glucose tolerance Test

Biweekly 2 g/kg intraperitoneal glucose tolerance test (IPGTT) were performed after rats were fasted overnight. IPGTT assessments were conducted using a 45% glucose solution (Corning, Manassas, VA) diluted to a 20% solution using normal saline and given to fasted rats at a dose of 2 g/kg. Capillary blood glucose levels were obtained via tail prick using a standard glucometer (Contour, Bayer, Mishawaka, IN) prior to injection and at 10, 30, 60, 90, 120, and 150 minutes.

### Histology

At the end of the study period, rats were sacrificed under isoflurane (Pirmal Critical Care, Bethleham, PA) anesthesia. Blood and tissue were obtained. Rat pancreases were weighed, measured and a small 0.2×0.2 cm section was cut from the pancreas tail for biochemical and histological assessments. Rat pancreas sections (5 µm thick) were fixed in 10% buffered formalin, embedded in paraffin blocks, and sectioned. The pancreas tissue was processed for hematoxylin-eosin (H&E) staining using standard techniques. Using Schmidt criteria, interstitial edema, leukocyte infiltration, acinar cell destruction, and total scores were evaluated individually by two pathologists blinded to the source of the histology sections they evaluated. Pancreatitis was scored using a quantitative grading system as described by Schmidt et al [14]. Classification (Table 1) is based on the presence of edema, leukocyte infiltration, acinar cell necrosis, and hemorrhage.

**Table 1:** Quantitative grading score for pancreatitis.

Score	0	1	2	3
Interstitial edema	none	Interlobular	Lobule involved	Isolated island like acinar cells
Leukocyte infiltration	none	<20%	20–50%	>50%
Acinar cell necrosis	none	<5%	5–20%	>20%
Hemorrhage	none	<5%	5–20%	>20%

### Pancreatic malondialdehyde content (MDA)

Pancreatic MDA was measured by thiobarbituric acid colorimetric method using MDA assay kit (Cayman Chemical Company, Ann Arbor, MI). Each pancreas tissue (100 mg) was placed in lysis buffer (RIPA, Thermo Scientific, Piscataway, NJ) and homogenized using a hand held Homogenizer (Power Gen, Fischer Scientific).

Samples were then centrifuged at 1200 RPM at 4°C. The absorbance of the supernatant was measured by spectrophotometry at 535 nm for MDA content, as MDA reacted with thiobarbituric acid and turned pink after a 1 hour boil. The MDA concentration was calculated from the standard curve and expressed as µM.

### Pancreatic Myeloperoxidase (MPO) content

Pancreatic MPO was measured using a standard colorimetric ELISA kit (BioVision, Milpitas, CA). Each pancreatic tissue (100 mg) was placed in MPO assay buffer (Bio Vision) and homogenized using a hand held Homogenizer (Power Gen, Fischer Scientific). Samples were then centrifuged at 13,000 G at 4°C. Supernatant (50 µl) at a 1∶100 dilution was added to the reaction mix and incubated for 30 minutes at 25°C. The absorbance of the supernatant was measured by spectrophotometry at 412 nM. The MPO concentration was calculated from the standard curve. MPO activity is reported as nmole/min/ml  =  mU/ml. One unit of MPO activity is defined as the amount of enzyme that hydrolyzes the substrate and generates taurine chloramine to consume 1.0 µmole of TNB per minute.

### Islet isolation and assessment of endocrine function

After removing and weighing the pancreas, the pancreatic duct was cannulated and a small piece of plastic cannula was sutured into the pancreatic duct. Pancreatic islet isolation was carried out as described previously [15], [16]. Briefly, the plastic tubing attached to the pancreatic duct was accessed and 20 mg Collagenase V (Sigma-Aldrich, St. Louis, Mo., USA) was infused into the pancreas. The pancreases of 4 rats per group were harvested, digested and islets were isolated by passing the tissue through a 500 µm mesh filter using Ficoll density solution. The islets were stained with dithizone and counted to estimate the islet equivalent (IEQ). Islet samples were transferred to 35 mm Petri dish with grid filled with1 mL of PBS and 10 drops of dithizone. The petri dish with islet sample was placed on inverted microscope to count. There is a scale in the left eye piece with each tick mark representing 25 µm when using the 4× objective. Islets of varying diameters are normalized to a number of Islet Equivalents of 150 µm diameter by mathematically compensating for their volumes. An islet with 150 um in diameter is counted as 1 IEQ [17], [18].

### Assessment of islet viability

The cultured islets were dissociated into single cell suspensions using the previously described method [19]. Aliquots of 700 IEQ were re-suspended in 1 mL accutase (Innovative Cell Technologies, Inc, San Diego, CA) in a 15-mL tube, incubated at 37°C for 10 minutes, and then dispersed by gentle pipetting. Digestion was stopped with 1 mL cold newborn calf serum (NCS, HyClone labs, Logan, UT). Washed Cells were transferred into filtered flow cytometry (FACS) tubes (BD Falcon, Franklin Lakes, NJ) to remove undigested tissue. The cells were then stained with 100 ng/mL of tetramethylrhodamine ethyl ester (TMRE; Molecular Probes) for 30 min at 37°C in Phosphate Buffered Saline. TMRE selectively binds to mitochondrial membranes, allowing for detection of apoptosis. After washing, cells were stained with 7-aminoactinomycin D (7-AAD; Molecular Probes), which binds to DNA when cell membrane permeability is altered after cell death. Analysis was performed by flow cytometry (Accuri C6 cytometers, Ann Arbor, MI, BD Bioscience) Acurri (Accuri C6 software, San Jose, CA) software was used for analysis.

### Assessment of alpha and beta ratio

The experiment was accomplished similarly to the above protocol prior to staining. Single cell suspensions were placed in 4% paraformaldehyde for 10 minutes at room temperature. After successive PBS (Corning, Manassas, VA) washes, the cells were stained with chicken polyclonal antibody to insulin (1∶100; Abcam Inc., Cambridge, MA) and mouse monoclonal antibody to glucagon (1∶100, Sigma, St. Louis, MO). Secondary antibodies were then applied, with goat anti-chicken (1: 200; Alexa Fluor 488 goat anti-chicken IgG, Life Technologies, Grand Island, NY) and goat anti-mouse (1: 200; Alexa Fluor 647 goat anti-chicken IgG, Life Technologies, Grand Island, NY). Omission of the primary antibody served as negative control. Analysis was performed by flow cytometry (Accuri C6 cytometers, Ann Arbor, MI, BD Bioscience) Acurri (Accuri C6 software, San Jose, CA) software was used for analysis.

### In vitro human pancreatic tissue studies

*In vitro* studies were performed on human pancreatic tissue obtained from NIH/JDRF sponsored integrated islet distribution program (IIDP, https://iidp.coh.org/) or from collaborators. Institutional Review Board exemption was obtained at University of California Irvine. Our study does not involve any information including living individuals, identifiable and private information.

Human pancreatic tissues were obtained after removal of islet cells. Human non-endocrine pancreatic tissue was treated with DMF at preconditioned concentrations dissolved in dimethyl sulfoxide (Sigma, St. Louis, MO). Human pancreatic tissue was cultured in Roswell Park Memorial Institute (RPMI-1640) solution containing additionally 10% NCS and Penicillin 100 U/ml/Streptomycin 50 µg/ml (Corning, Manassas, VA).

Human tissue was cultured with or without DMF. After a culture period of twenty four hours the tissue was homogenized (Power Gen 125, Fischer Scientific, Pittsburg, PA) with Lysis Buffer (RIPA, Thermo Scientific, Piscataway, NJ). Protein aliquots (45 µg) were mixed with sample buffer (Bio-Rad laboratories, Richmond, CA) boiled for 5 minutes and separated via 4–12% BIS TRIS (2-[Bis(2-hydroxyethyl)amino]-2-(hydroxymethyl)-1, 3-propanediol) (Life Technologies, Carlsbad, CA). After transfer to nitrocellulose membranes (Biorad) the membranes were blocked with PBS/0.1%Tween containing 2% dry milk for 90 minutes at room temperature. The membranes were then probed with mouse monoclonal antibody to heme oxygenase (HO-1) (1∶1000) and mouse monoclonal antibody to β-actin (1∶1000) (Cell Signaling, Danvers, MA) followed by rabbit anti-mouse IgG (1∶3000). The membranes were developed with an enhanced chemoluminescence detection kit (Bio-Rad) and exposed to X-ray film (Kodak, Rochester, NY). To evaluate the results of the western blot, the relative intensities of individual bands were determined with ImageQuant TL 7.0 (GE healthcare Life Sciences, Pittsburg, PA).

### Statistical Analysis

Student's ***t*** test and one-way analysis of variance (ANOVA) were used in statistical analysis of the data using Excel for Windows software (Microsoft, Redmond, WA). P values equal to or less than 0.05 were considered significant. Data are expressed as mean ± SD.

## Results

### Effects of DMF on body weight in L-Arginine-induced CP

**Figure 1:**
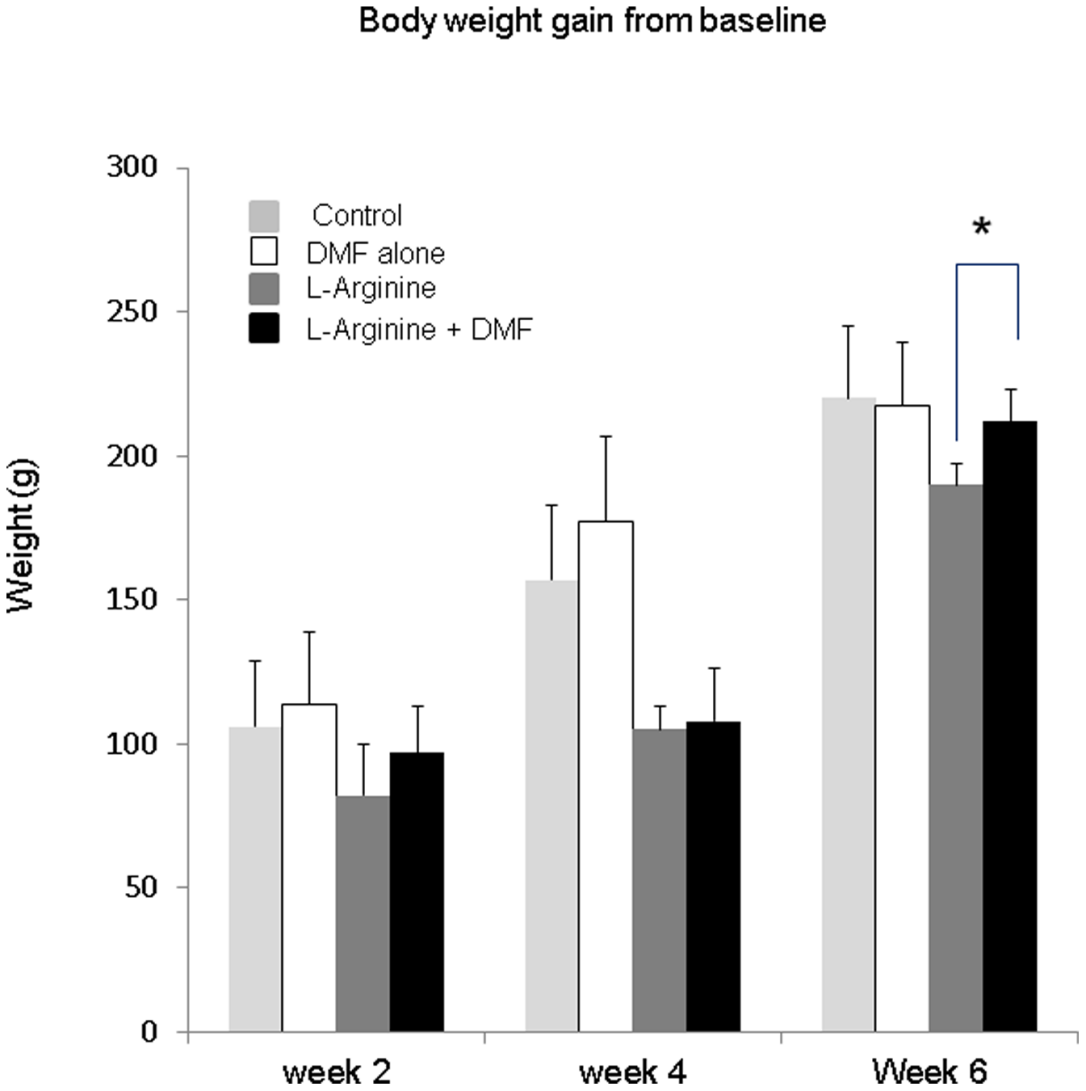
Average change in body weight. Body weights were similar for all four experimental groups: control, DMF-alone, L-Arginine, and L-Arginine + DMF (n = 3, 3, 6, 6) at baseline and weeks 2, 4 and 6. There was a significant difference of the change in baseline weight at 6 weeks between L-Arginine and L-Arginine + DMF. * indicates a significant p<0.05 value. Data represent Mean ±S.D.

The average body weights for control, DMF alone treated, L-Arginine vehicle treated, and L-Arginine with DMF treated rats were similar at baseline and at three and six weeks during the experiment. Compared to baseline the average weight gain in the L-Arginine + DMF treated group was similar to that found in the control and DMF alone treated rats. However, average weight gain was significantly reduced in the L-Arginine alone-treated group (P<0.05) at 6 weeks (Figure 1).

### Treatment with DMF improved glucose tolerance in rats with L-Arginine- induced CP

**Figure 2:**
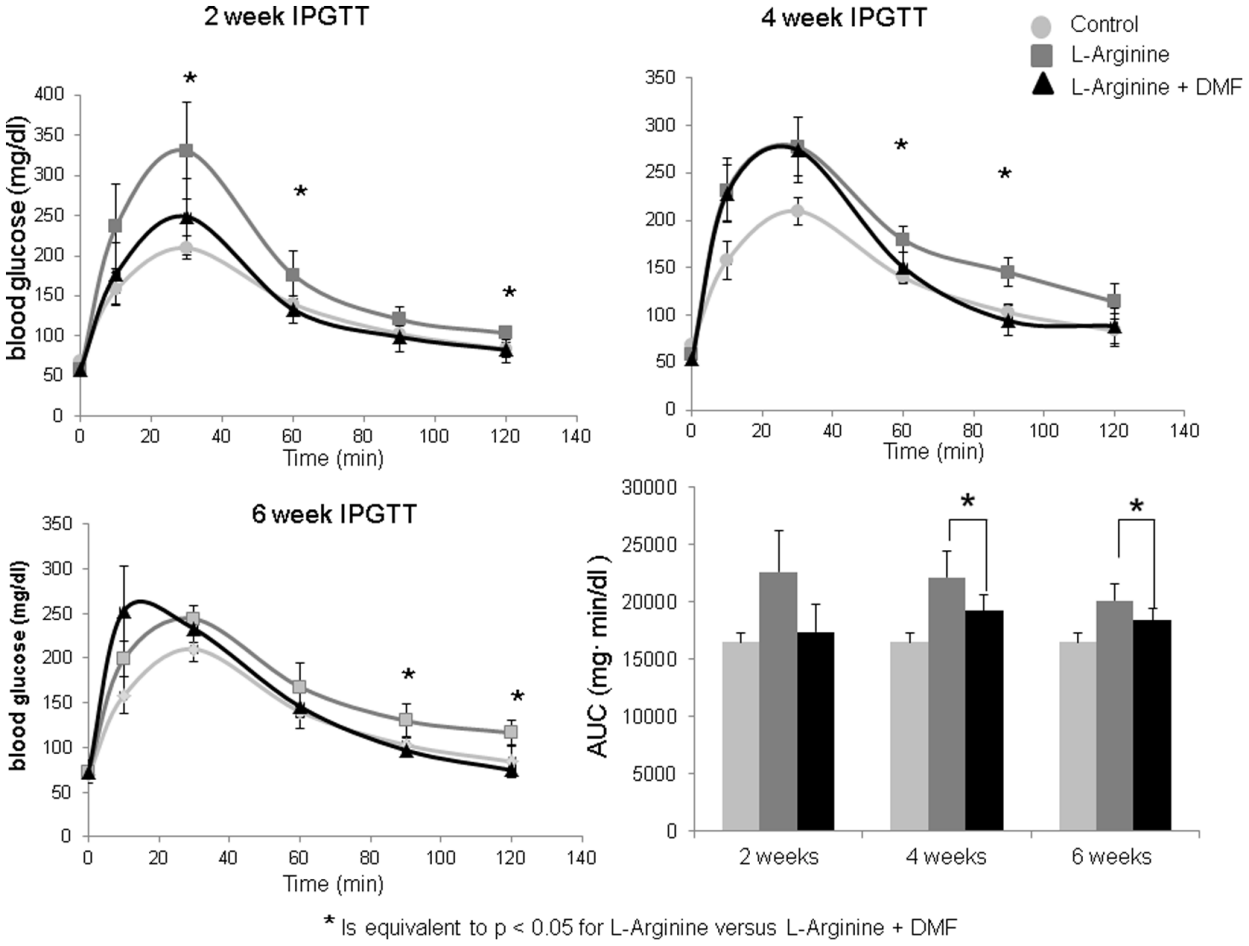
IPGTT at 2, 4, and 6 weeks. IPGTT conducted at 2 weeks reveals a significant change in the blood glucose level for L-Arginine (n = 6) and L-Arginine + DMF group (n = 6) at 30 and 60 minutes. At 4 weeks the differences between the two groups were seen at 60 and 90 minutes. Lastly, at 6 weeks there was a significant change between L-Arginine and L-Arginine + DMF group at 90 and 120 minutes. These differences indicate L-Arginine had a slower return to baseline glucose levels compared to the rats supplemented with DMF. An * indicates a significant differences with P<0.05. AUC was calculated. L-Arginine + DMF had a statistically lower blood glucose AUC compared to L-Arginine alone and results were similar to control rats for all weeks. * is equivalent to P<0.05 for L-Arginine versus L-Arginine + DMF.

Intra-peritoneal glucose tolerance test performed at two, four, and six weeks revealed significant impairment in the untreated and significant improvement in the DMF-treated group at several time points (Figure 2). Moreover, the area under the curve (AUC) for the IPGTT revealed significantly improved glucose tolerance, as seen with lower blood glucoses, in the DMF treated rats (Figure 2).

### Treatment with DMF improved pancreas pathology in rats with L-Arginine-induced CP

**Figure 3:**
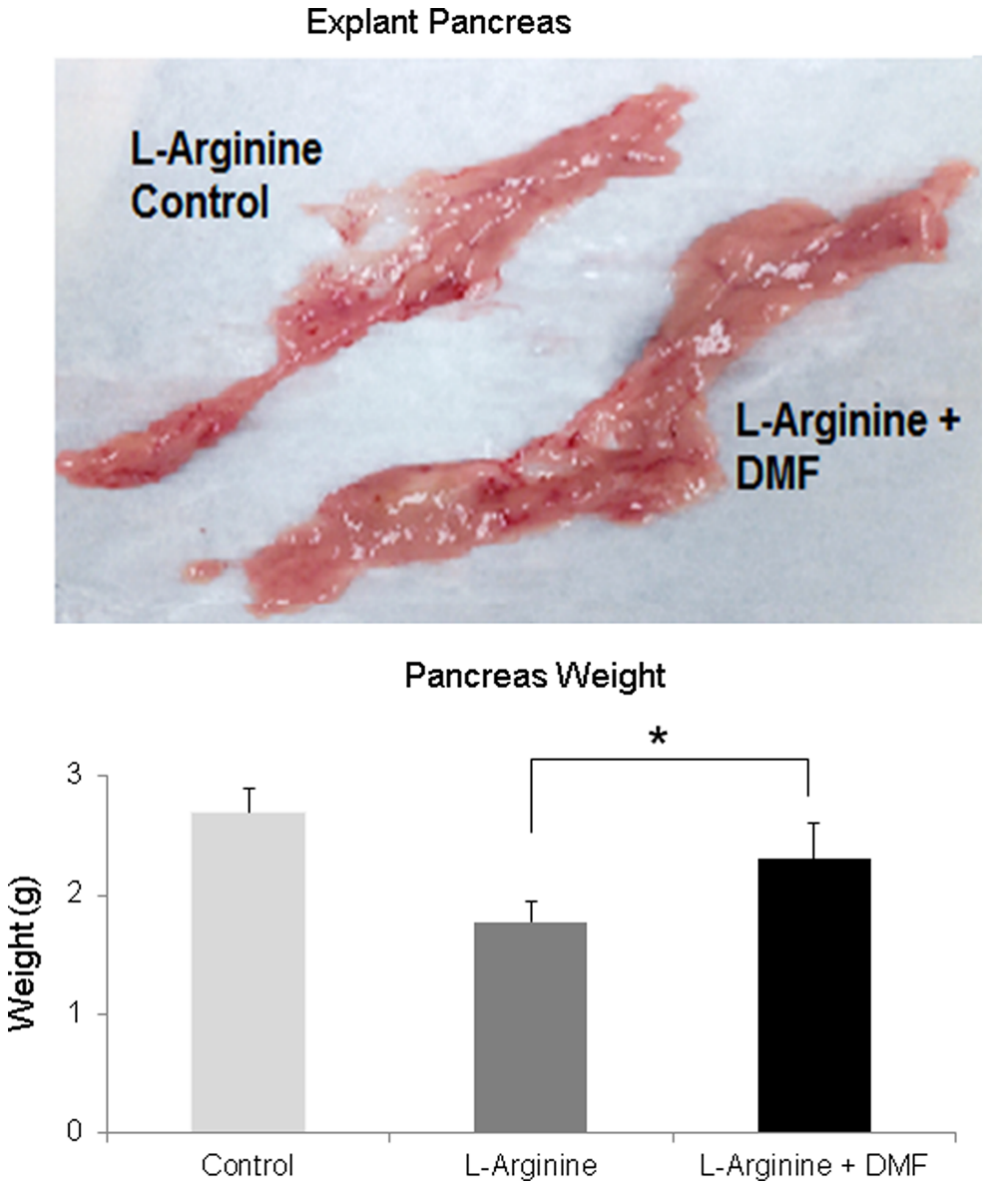
Explant pancreas weight. At explantation, pancreases from the control, L-Arginine and L-Arginine+ DMF rats were weighed at the end of 7 weeks. Results revealed a statistically larger pancreas weight (g) in the L-Arginine + DMF group. The image above shows an atrophic pancreas in the L-Arginine group compared to the L-Arginine + DMF group. The bar graphs demonstrated an average of 6 total L-Arginine rats and 6 L-Arginine + DMF rats. * indicates a significant p<0.05 value. Data represent Mean ±S.D.

On explanation, following 7 weeks of L-Arginine induction, the pancreas of the L-Arginine alone treated rats was visibly atrophic and had a lower weight (g) compared to the L-Arginine + DMF treated rats (p<0.05) (Figure 3). Pancreases of the DMF alone treated rats appeared grossly similar to the control rats' pancreas (Data not shown).

**Figure 4:**
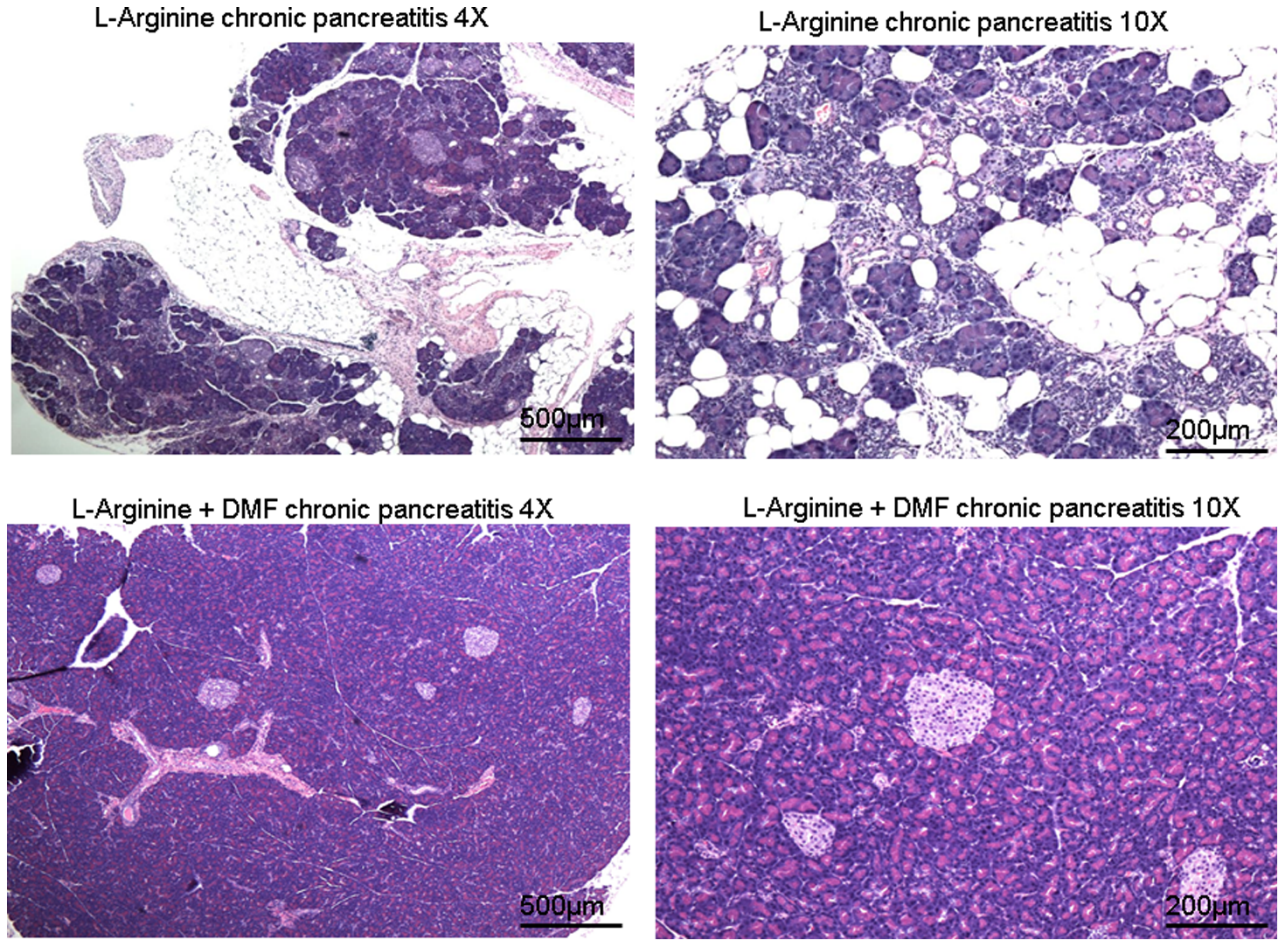
L-arginine induced chronic pancreatitis in a rodent model. Representative photomicrograph of an H&E stained section of pancreas after 7 weeks of chronic L-Arginine induction. L-Arginine + DMF treated rats had significantly reduced severity of destruction of acinar architecture, perilobular edema, and infiltration of fat.

**Figure 5:**
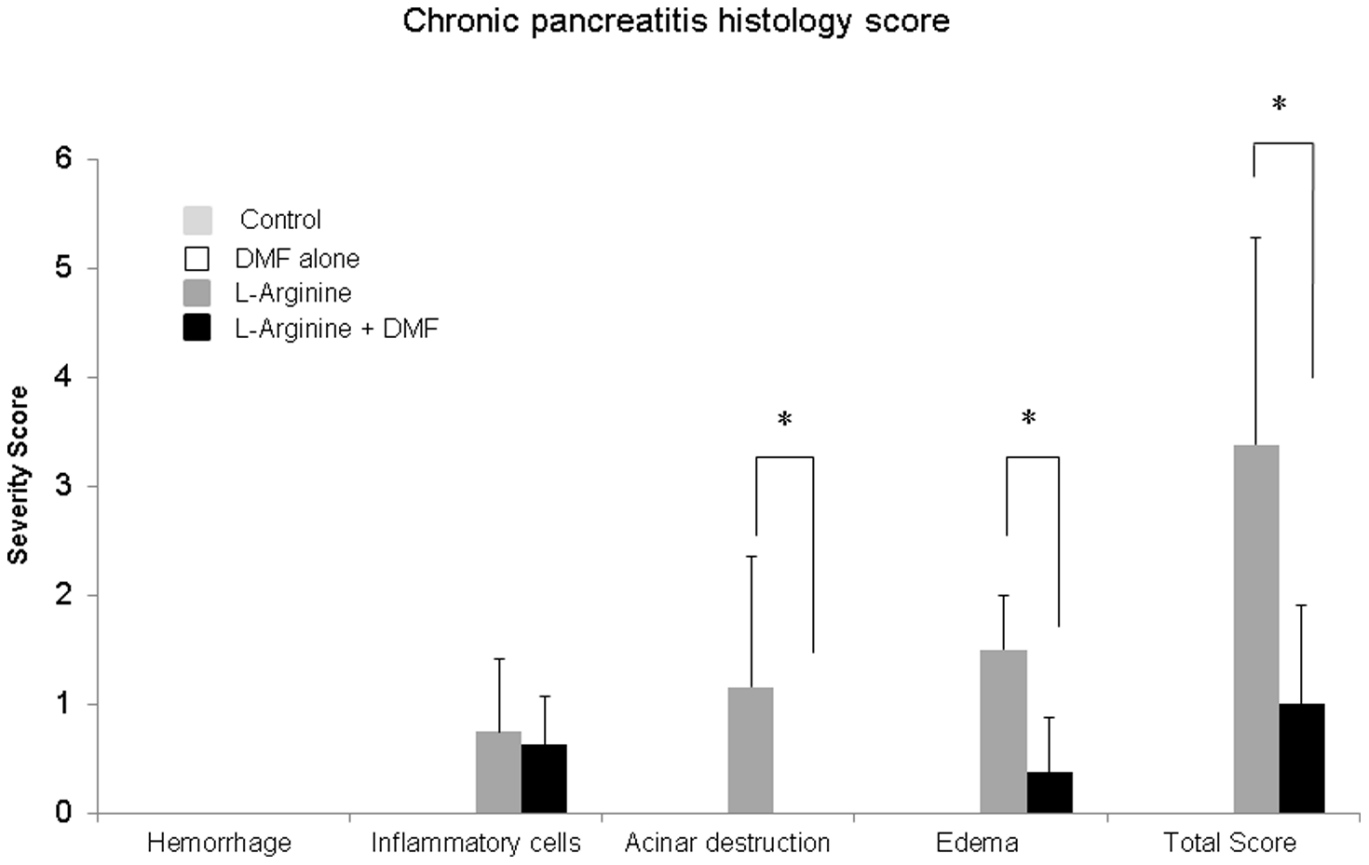
Quantitative chronic pancreatitis Score: hemorrhage, interstitial edema, leukocyte infiltration, acinar cell destruction, and average in total pancreatitis score. Histological pancreas slides were evaluated by two pathologists blinded to the source of the histology sections and given a score based on severity from 0–3 based on four criteria: hemorrhage, inflammatory cell infiltration, acinar destruction, and edema. The total score was also calculated. Based on quantitative scores for all four criteria there was a significantly higher severity score for L-arginine compared to L-arginine + DMF group. The severity scores in the normal and DMF alone treated rats were 0. (* indicates a significant p<0.05 value for L-arginine versus L-Arginine + DMF).

Administration of L-Arginine in rats caused significant histological changes in the pancreas including severe acinar architectural damage, edema, and fatty infiltration. Treatment with oral DMF resulted in significant (p<0.05) reductions in acinar architectural damage, edema, and fatty infiltration (Figure 4&5). Moreover, the severity score determined by two pathologists who were blinded to the source of the histology sections, was significantly higher in the L-Arginine alone rats compared to the L-Arginine + DMF rats (p<0.05) (Figure 5, Table 2). The severity scores in the normal and DMF alone treated rats were 0. Although, L-arginine has a mechanism of action that remains unclear there is evidence to support that L-arginine exerts its effects by producing oxidative stress and accumulation of nitric oxide *in vivo* [20]. DMF was extremely effective in protecting the treated rats against L-arginine induced chronic pancreatic changes most likely from the reduction in oxidative stress.

**Table 2:** Chronic pancreatitis histology score.

Groups	Inflammatory Cells	Acinar destruction	Edema	Hemorrhage	Total score
Control	0	0	0	0	0
DMF alone	0	0	0	0	0
L-arginine	0.75±0.66	1.16±1.2	1.5±0.5	0	3.38±2.36
L-arginine + DMF	0.625±0.45	0	0.375±0.5	0	1±0.95

### Improved biochemical parameters were seen in pancreatic tissue of rats treated with DMF

**Figure 6:**
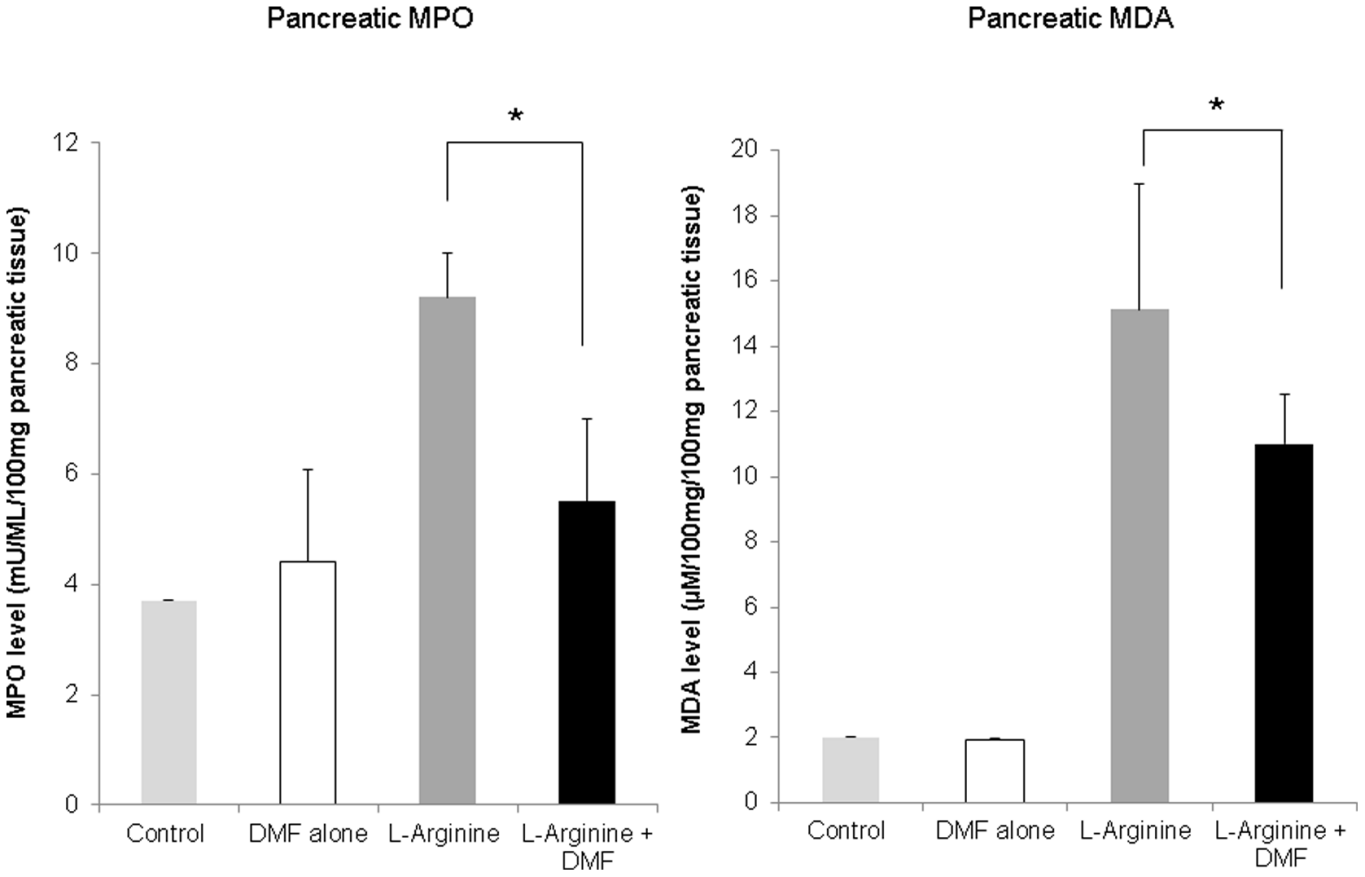
Effects of DMF on pancreatic MDA and MPO. MDA and MPO level in pancreatic tissue were determined after 7 weeks of chronic L-Arginine induction. There was a significant reduction of both pancreatic MDA and MPO levels in the L-Arginine + DMF treated rats (n = 3) compared to the L-Arginine alone treated rats (n = 3). There were no effects of DMF observed on MDA and MPO level under the condition of no L-Arginine induction. * indicates a significant p<0.05 value. Data represent Mean ±S.D.

MPO catalyzes conversion of chloride and hydrogen peroxide to hypochlorite and is secreted actively by neutrophils during inflammatory conditions [21]. MPO was measured as a marker of inflammatory stress. MDA is a byproduct of lipid peroxidation and a marker of cellular damage and oxidative stress. Both the pancreatic MPO and MDA levels were significantly higher in the L-Arginine than in the control group and were significantly lower in the L-Arginine + DMF treated rats compared to the L-Arginine alone rats (P<0.05)(Figure 6). The observed reduction of MPO and MDA levels demonstrates the efficacy of DMF in attenuating oxidative and inflammatory stress in this model.

**Figure 7:**
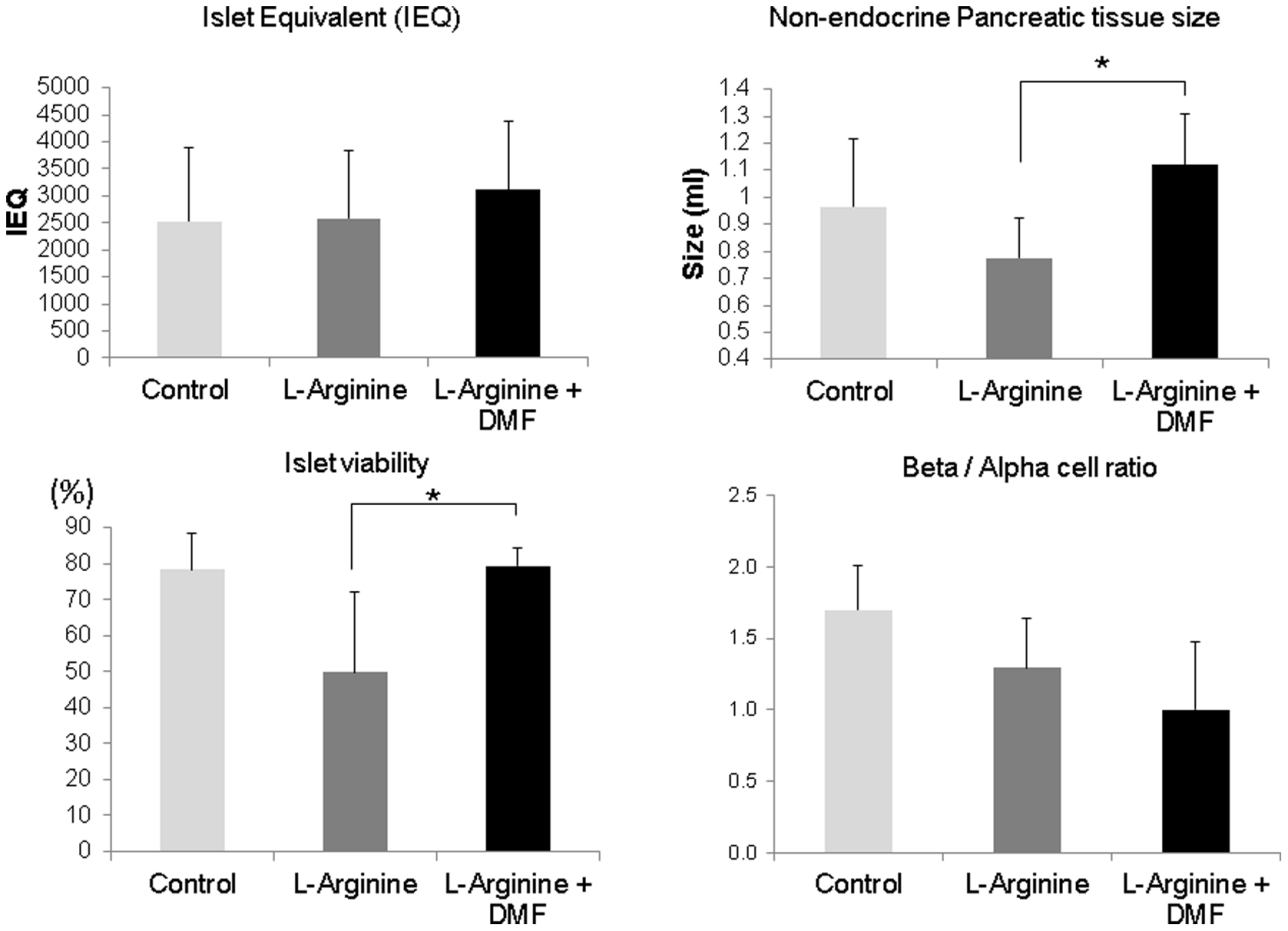
Endocrine and non-endocrine cells characteristics after isolation. After isolation of islets, the islet number was found to be similar between L-Arginine (n = 6) and L-arginine + DMF group (n = 6), however, the pancreatic tissue volume remaining after extraction of islets was significantly larger in the L-Arginine + DMF group compared to the L-Arginine alone group. Quantitatively, the islet number was similar among all groups. Islet viability in the L-Arginine + DMF group was significantly higher but beta/alpha ratio was similar. * indicates a significant p<0.05 value. Data represent Mean ±S.D.

Islets from each experimental group were isolated and compared. The pancreatic tissue volume (mainly acinar cells) was significantly larger in the L-Arginine + DMF group than in the L-Arginine alone group (Figure 7). The smaller non-endocrine pellet corresponds to the atrophy observed on the gross examination of pancreas in the L-Arginine alone group. Quantitatively, the number of islets was similar among all groups; however, the islets had improved viability on flow cytometry in the L-Arginine + DMF group compared to the L-Arginine alone group. The beta/alpha ratio appeared lower in the L-Arginine alone group but the difference did not reach statistical significance (Figure 7). The islets from the DMF treated rats were resistant to cell death and apoptosis *in vitro* which may account for the improved glucose tolerance in the rats *in vivo*.

### HO-1 was upregulated in human pancreatic tissue treated with DMF in vitro

**Figure 8:**
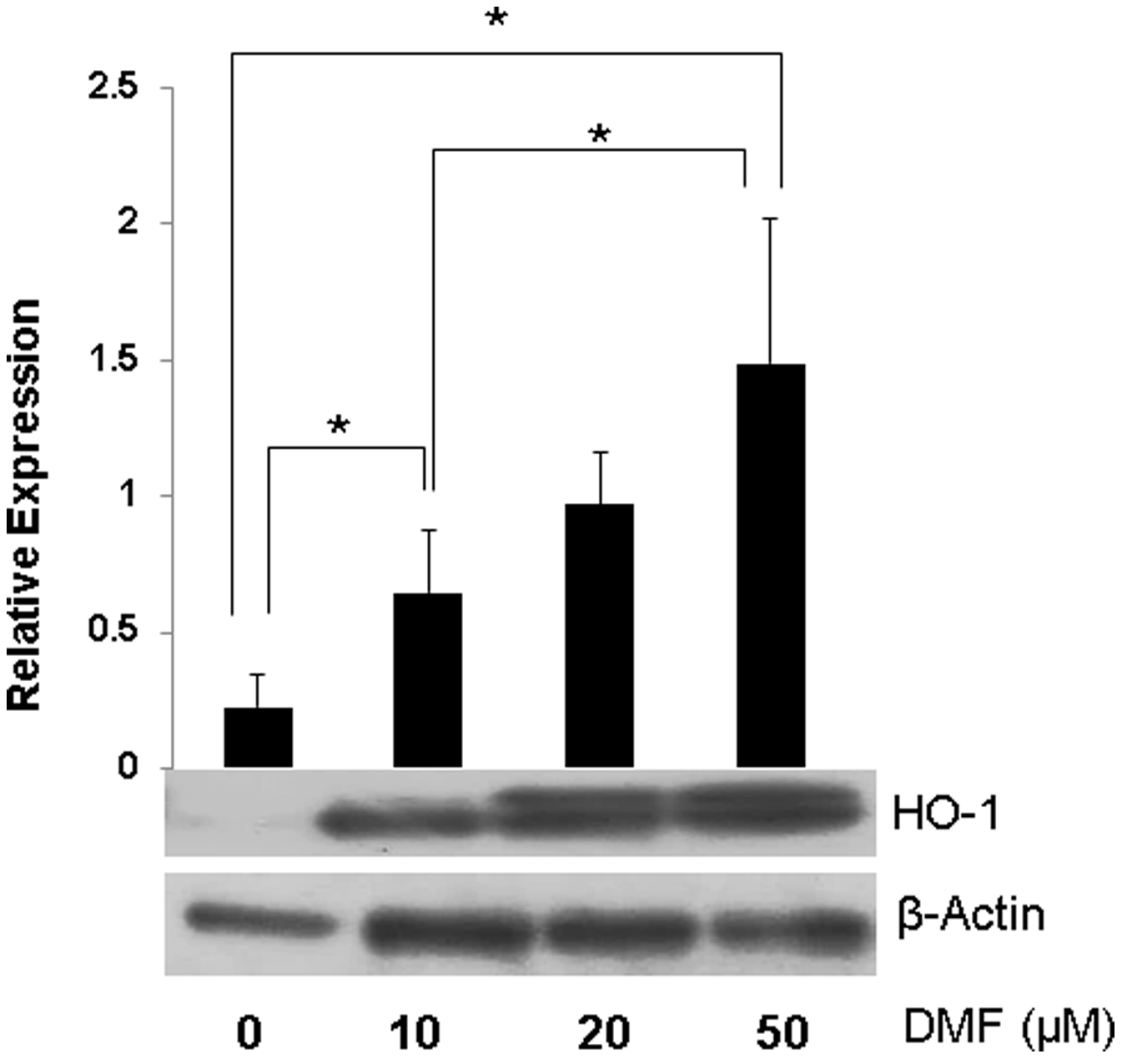
Effects of DMF on HO-1 expression in human pancreatic tissue. Representative western blots of HO-1. The bar graph summarizes the western blot data. DMF significantly enhanced protein expression of HO-1 in a dose dependent manner. Data are representative of four experiments using human islet preparations from independent donors and represent Mean ±S.D. * indicates a significant p<0.05 value.

To explore the effect of DMF on expression of the pancreatic antioxidant enzyme HO-1, Western blot was performed 24 hours after incubation of pancreatic tissue with and without media containing DMF. Western blot revealed a significant increase in the HO-1 expression in DMF-treated human islets (Figure 8). Enhanced production of the key antioxidant enzyme in pancreatic tissue treated with DMF supports the hypothesis that DMF ameliorates oxidative stress through regulation of anti-oxidative enzymes.

## Discussion

Due to the significant life changing processes that affect patients with CP, it is imperative that more effective treatments are explored. The results of this study revealed a pronounced difference in the pathology, histology, and pancreatic biochemical markers between the DMF treated animals and those given the vehicle alone. Our data indicates an overall improvement in the inflammatory and oxidative stress in the DMF treated animals as evidenced by the lowered levels of MDA and MPO. Although, fibrosis is a prominent feature of clinical chronic pancreatitis, we did not appreciate significant fibrosis in our model. Our findings were similar to the results achieved by Yamaguchi et al [13] in which the destroyed pancreas was replaced by fatty tissue instead. Although this model does not fully resemble clinical CP, the use of L-arginine-induced CP in this study was effective in determining the effects of DMF on protection of islets and non-endocrine pancreatic tissue in experimental CP. Although our islets appeared histologically normal and similar islet yield was obtained, islet cell viability was significantly impaired in vehicle-treated and improved with DMF administration.

It is postulated that the anti-oxidant effects of DMF gave the vulnerable islets the fortitude to withstand the stress of chronic L-Arginine-induced inflammation and the stress of tissue isolation. Given this finding, one would expect to see improved glucose tolerance tests as was observed. Enhanced glucose tolerance was observed by the DMF treatment. This observation suggest that by attenuating oxidative stress and fortifying the antioxidant capacity, DMF was effective in improve glucose homeostasis *in vivo*.

This phenomenon was also replicated in a study by Weaver et al. who established a CP model in a rat by daily L-Arginine injections for up to four weeks. After four weeks only single isolated acini remained within connective tissue however normal islets were seen histologically. Despite histologically normal islets, impairments in glucose homeostasis were seen in rats given chronic L-arginine injections, with significant changes seen on glucose tolerance tests between weeks two through four [22].

The endocrine pancreas develops as a highly organized microvascular network with elegant capillary fenestrations largely dependent on vascular endothelial growth factor (VEGF). Although, normal appearing islets were seen histologically in our study, severe destruction of neighboring acini were encountered and it is postulated that this highly organized capillary network was also disrupted. In an elegant study, Kostromina and associate showed that knockout mice of the signal transducer and activator transcription 3 (STAT-3), a transcription factor regulating VEGF, exhibited glucose intolerance and impaired insulin secretion *in vivo* but normal insulin secretion and equivalence in isolated islets *in vitro* [23].

Moreover, it has been established that beta cells are particularly vulnerable to oxidative stress. Endogenous antioxidant enzymes existing within host tissues primarily protect from excessive levels of reactive oxygen species. An important consideration would be the level of the host's antioxidant defenses within the cells. Islets are scattered throughout the whole pancreas and comprise only 1–2% of pancreatic tissue while acinar cells comprise 80–85% in the pancreas. It has been reported that the islets have very low level of intrinsic antioxidant enzyme expression and activity, including superoxide dismutase, catalase, and glutathione peroxidase [24]. However, the level of antioxidant enzyme expression in each type of human pancreatic endocrine and exocrine cells has yet to be reported. In previous experiments, (unpublished data) we have found beta cells to have a particularly low level of antioxidant enzymes compared to alpha cells. It is therefore, reasonable to deduce that improving the endogenous antioxidant capacity of the rats would enhance the viability of the islets exposed to chronic oxidant stress and improve glucose tolerance as was observed in this study.

Dimethyl Fumarate has a unique antioxidant and anti-inflammatory spectrum. Although the mechanism of DMF action remains unidentified, its low side effect profile has enabled its use and repurposing for over 20 years. Fumaric acid esters were first employed in the treatment of psoriasis under the trade name Fumaderm. This was based on the anti-proliferative effect of this compound on lymphocytes. Later reports found that DMF selectively up-regulated Th2 cytokines and suppressed a Th1 response [7], [25], [26]. Subsequent studies showed that DMF reduces expression of genes encoding pro-inflammatory cytokines and chemokines, and increases expression of anti-oxidant molecules [26], [27] – effects likely to contribute to its antipsoriasis efficacy. These findings have led to increased interest in using DMF in treatment of other auto-immune or inflammatory diseases, including multiple sclerosis (MS). Recently, clinical trials demonstrated that in patients with multiple sclerosis, DMF (BG-12) significantly reduced the proportion of patients who had a relapse, the annualized relapse rate, the rate of disability progression, and the number of lesions on MRI without significant adverse events [28]. These findings led to its FDA approval for first line oral therapy in MS patients on March 2013. Although clinically the exact mechanism of action of DMF has not been determined, DMF has been shown to induce expression of the potent endogenous antioxidant enzyme, HO-1 in experimental setting [29]. We were able to replicate these findings with *in vitro* assessments of HO-1 expression in DMF treated human tissue. HO-1 is a ubiquitously expressed antioxidant that is inducible in response to oxidative stress [30]. The upregulation of HO-1 in our experiment is a likely mechanism, among others, that lead to DMF's protective effects. Others have also implicated that DMF exerts its antioxidant effects as an Nrf2 activator. The pathway plays a critical role in the induction of genes that encode numerous antioxidants and detoxifying enzymes [31], [32]. More likely, DMF has multiple mechanisms that afford its potent antioxidant efficacy.

In conclusion, long-term treatment with DMF was effective in ameliorating the histological lesions and biochemical abnormalities and improving beta cell function in our rodent model of L-Arginine-induced CP. Although, pain was not assessed in this study the authors wish to study experimental pain syndromes and DMF in future experiments given the wealth of knowledge that connects chronic pain with inflammation. Further research is needed to determine the efficacy of this drug for the treatment of CP in humans.
